# Supplementary material for: EPicker is an exemplar-based continual learning approach for knowledge accumulation in cryoEM particle picking
Source: Nat Commun. 2022 May 5;13:2468. doi: 10.1038/s41467-022-29994-y (PMC9072698; doi:10.1038/s41467-022-29994-y)
Supplement: Supplementary file 1 — Supplementary Information [file 41467_2022_29994_MOESM1_ESM.pdf]

Supplementary Information for:

EPicker is an exemplar-based continual learning approach for knowledge accumulation in cryoEM particle picking.

Authors: Xinyu Zhang, Tianfang Zhao, Jiansheng Chen, Yuan Shen, Xueming Li

Inventory of Supporting Information:

Supplementary Table 1 2
Supplementary Table 2 3
Supplementary Table 3 4
Supplementary Table 4 5
Supplementary Table 5 6
Supplementary Table 6 7
Supplementary Table 7 8
Supplementary Table 8 9
Supplementary Table 9 10
Supplementary Figure 1 11
Supplementary Figure 2 12
Supplementary Figure 3 13
Supplementary Figure 4 14
Supplementary Figure 5 15
Supplementary Figure 6 16
Supplementary References 19

Supplementary Tables

Supplementary Table 1. Comparison of different weighting factors for continual learning. An initial model was trained on micrographs from five EMPIAR datasets: 1–5. Then, EMPIAR-10017 was used for continual learning based on the initial model. EPicker chose weighting factors $\lambda_d = 0.1$ and $\lambda_r = 0.01$, which obtained the highest mAP and mAR. The cell with bold value indicates that the corresponding strategy achieves the highest performance.

Strategy	E10089 (1)	E10146 (2)	E10028 (3)	E10203 (4)	E10025 (5)	E10017 (6)	mAP/mAR
$\lambda_d = 1, \lambda_r = 0.1$	96.8/99.2	95.0/97.5	96.8/98.0	90.8/98.3	90.6/95.5	94.2/97.1	94.0 /97.6
$\lambda_d = 0.1, \lambda_r = 0.01$	96.4/99.2	94.9/97.2	96.4/97.7	90.2/98.3	90.3/95.5	96.2/98.5	**94.1/97.7**
$\lambda_d = 0.01, \lambda_r = 0.001$	95.5/98.8	94.2/96.8	96.1/97.3	89.2/97.0	90.0/95.1	96.8/99.0	93.6/97.3

Supplementary Table 2. Impact of three components of loss function in EPicker. An initial model was trained on micrographs from five EMPIAR datasets: 1–5. EMPIAR-10017 was used for continuous learning based on the initial model. Using only the object detection loss term, an mAP of 92.3% was achieved. Adding only the knowledge distillation or regularization loss term resulted in mAP improvements of 1% and 0.4%, respectively. Combining all three components resulted in the highest mAP and mAR. Cells with bold values indicate that the corresponding loss function achieves the highest performance.

Loss function	E10089 (1)	E10146 (2)	E10028 (3)	E10203 (4)	E10025 (5)	E10017 (6)	mAP/mAR
L_{OD}	92.2/98.3	93.1/96.5	96.0/97.3	85.9/97.7	89.8/95.1	96.8/98.9	92.3/97.3
$L_{OD} + L_{Distill}$	93.7/98.3	94.3/97.1	96.2/97.3	88.4/98.9	89.8/95.2	**97.3/99.0**	93.3/97.6
$L_{OD} + L_{Reg}$	92.3/98.1	94.0/96.7	95.9/97.5	88.6/98.9	88.7/94.6	96.6/98.9	92.7/97.5
$L_{OD} + L_{Distill} + L_{Reg}$	**96.4/99.2**	**94.9/97.2**	**96.4/97.7**	**90.2/98.3**	**90.3/95.5**	96.2/98.5	**94.1/97.7**

Supplementary Table 3. Comparison of different continual learning methods. The results of joint training (JT) on datasets 1–5 and 1–6 were calculated as the upper bounds of performance. For continual learning, an initial model was trained on micrographs from five EMPIAR datasets: 1–5. EMPIAR-10017 was used for continuous learning based on the initial model. EPicker resulted in the highest mAP and mAR values. Cells with bold values indicate that the corresponding method achieves the highest performance.

Name<br>No.	E10089<br>(1)	E10146<br>(2)	E10028<br>(3)	E10203<br>(4)	E10025<br>(5)	E10017<br>(6)	mAP/mAR
JT(1-6)	97.5/99.1	96.8/97.5	97.6/98.0	95.3/99.4	92.0/96.7	97.0/99.5	96.0/98.4
JT(1-5)	97.5/99.1	96.4/97.2	96.7/97.5	93.1/98.9	92.0/96.6	-	95.1/97.9
MAS[1]($F_{extract}$)	92.7/98.8	92.8/96.0	95.2/97.2	88.8/98.9	89.3/94.9	**96.6/98.9**	92.6/97.5
MAS[1](F_{OD})	91.1/98.6	92.7/96.5	94.5/97.5	85.9/98.3	88.9/94.9	**96.6/98.9**	91.6/97.5
AD in Faster ILOD[2]	93.9/98.6	94.0/96.8	95.3/97.2	88.3/98.9	90.3/95.4	96.3/98.9	93.0/97.6
EPicker	**96.4/99.2**	**94.9/97.2**	**96.4/97.7**	**90.2/98.3**	**90.3/95.5**	96.2/98.5	**94.1/97.7**

Supplementary Table 4. Comparison of different feature extraction networks. AP/AR values based on the joint training models on six datasets. Two types of feature extraction networks, ResNet[3] and DLA[4], were tested. The numbers following the network names denote the number of layers used in the corresponding neural network. Cells with bold values indicate that the corresponding network achieves the highest performance.

Network	E10089 (1)	E10146 (2)	E10028 (3)	E10203 (4)	E10025 (5)	E10017 (6)	mAP/mAR
ResNet-18	97.6/99.1	**97.0/97.6**	96.8/97.7	94.1/97.7	89.3/94.6	92.6/95.8	94.6/97.1
ResNet-34	97.1/98.9	96.7/97.5	96.7/97.9	94.9/98.9	91.5/95.9	96.3/98.7	95.5/98.0
DLA-18	98.1/99.4	96.6/97.3	96.9/98.0	95.0/98.9	**92.2/96.7**	96.1/99.3	95.8/98.3
DLA-34	97.5/99.1	96.8/97.5	97.6/98.0	**95.3/99.4**	92.0/96.7	**97.0/99.5**	**96.0/98.4**
DLA-46	**97.6/99.2**	95.9/96.8	**97.6/98.6**	94.9/99.4	91.8/96.1	95.2/99.3	95.5/98.2

Supplementary Table 5. Comparison of training and picking times for different software. To evaluate the training process, we compared different software on a combination of 10 datasets, including 100 micrographs and 19300 annotated particles. All experiments were performed on a single GPU (RTX 2080Ti). The time of joint training on 10 datasets was measured for crYOLO, Topaz, and EPicker. For EPicker, the time of the continual learning was measured by adding the 10th dataset to a general model trained on the previous 9 datasets. To evaluate the picking process, the average time taken to pick a single micrograph was measured.

Software	Training time (100 micrographs)	Picking time (1 micrograph)
TOPAZ[5] (Joint Training)	30 min	0.5 s
crYOLO[6] (Joint Training)	56 min	0.3 s
DRPNet[7]	×	3 s
DeepCryoPicker[8]	×	30 s
EPicker (Joint Training)	50 min	0.3 s
EPicker (Continual Learning)	20 min	0.3 s

Supplementary Table 6. Evaluation results of EPicker trained on 10 datasets. The particles were manually picked and used for training or ground truth.

No.	EMPIAR dataset ID	AP/AR	Number of training/testing micrographs	Number of training/testing particles
1	E10017	96.9/99.3	10/5	5151/2963
2	E10025	91.5/96.4	10/5	6137/3364
3	E10028	97.7/98.0	10/5	1087/564
4	E10075	96.0/96.7	10/5	642/306
5	E10081	96.1/98.1	10/5	1380/620
6	E10089	96.1/98.0	10/5	1074/640
7	E10097	91.7/96.8	10/5	1460/598
8	E10146	97.0/97.5	10/5	1413/753
9	E10203	93.4/97.7	10/5	315/177
10	E10228	90.3/96.3	10/5	667/535

Supplementary Table 7. **Illustration of images in different datasets after local enlargement.** We selected 10 typical datasets from EMPIAR. In the continual learning process, different particles have significant differences in sizes and features, which are used to illustrate the generalization ability and robustness of EPicker.

E10089 (TcdA1)	E10146 (Apoferritin)	E10028 (80S ribosome)	E10203 (Nodavirus)	E10025 (20S Proteasome)
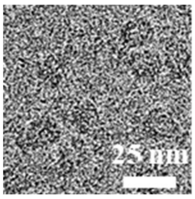	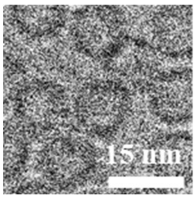	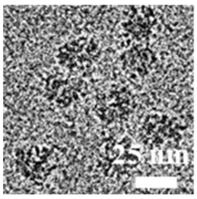	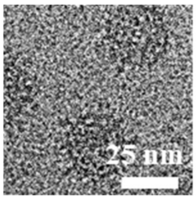	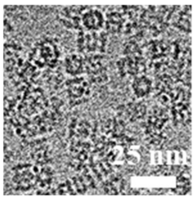
E10017 (Beta-galactosidase)	E10097 (Influenza hemagglutinin trimer)	E10075 (Phage MS2)	E10081 (HCN1 ion channel)	E10228 (Phosphodiesterase 6)
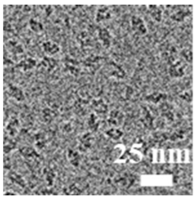	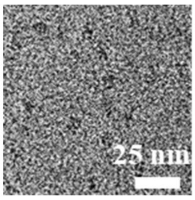	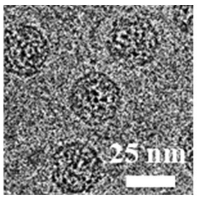	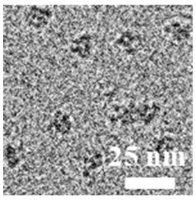	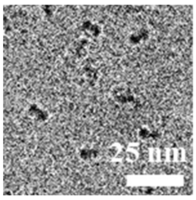

Supplementary Table 8. Comparison of different weighting factors for particle detection. Liposome dataset was used for training. EPicker predicted the size and the position of liposome simultaneously. EPicker chose the weighting factors $\lambda_{off} = 1$ and $\lambda_{size} = 0.1$, which obtained the highest mAP and mAR. The cell with bold value indicates that the corresponding strategy achieves the highest performance.

Strategy	$\lambda_{off} = 1, \lambda_{size} = 1$	$\lambda_{off} = 1, \lambda_{size} = 0.1$	$\lambda_{off} = 0.1, \lambda_{size} = 1$	$\lambda_{off} = 0.1, \lambda_{size} = 0.1$
Liposome	91.7/99.4	**92.8/99.4**	92.0/99.4	90.6/98.8

Supplementary Table 9. Datasets used to train the general model. The datasets without EMPIAR ID are from other unpublished resources.

No.	EMPIAR dataset ID	No.	EMPIAR dataset ID	No.	EMPIAR dataset ID
1	E10017	18	E10168	35	E10402
2	E10025	19	E10175	36	E10399
3	E10028	20	E10190	37	E10454
4	E10075	21	E10192	38	E10443
5	E10081	22	E10197	39	E10379
6	E10089	23	E10202	40	E10456
7	E10097	24	E10205	41	E10350
8	E10146	25	E10216	42	E10335
9	E10203	26	E10059	43	E10217
10	E10228	27	E10590	44	E10291
11	E10004	28	E10406	45	E10290
12	E10033	29	E10470	46	E10289
13	E10057	30	E10429	47	E10090
14	E10058	31	E10241	48	GspD
15	E10093	32	E10270	49	WLC
16	E10096	33	E10420	50	Nma111p
17	E10122	34	E10407	51	Fab

Supplementary Figures

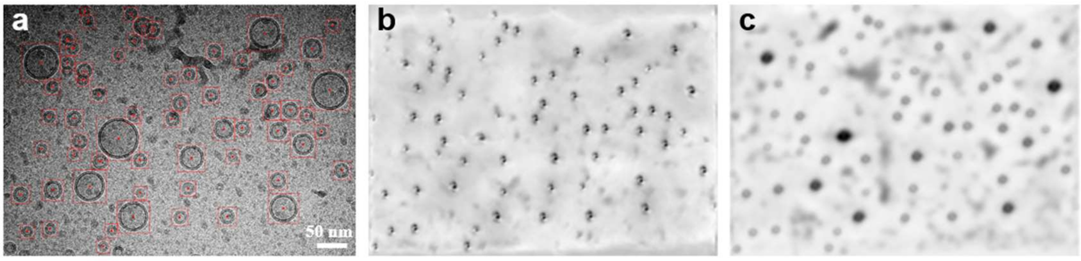

Supplementary Figure 1. Picking process for vesicles. The picking process for vesicles are similar to that for the particles (**Fig. 2**). The difference is that the size prediction is turned on. **a**) A typical micrograph of the liposome vesicles. The red squares indicate the picking result by EPicker, which shows both the position and size of each liposome. **b)** The heatmap for the center prediction. **c)** The heatmap for the size prediction. Both the heatmaps are generated by the object location sub-network.

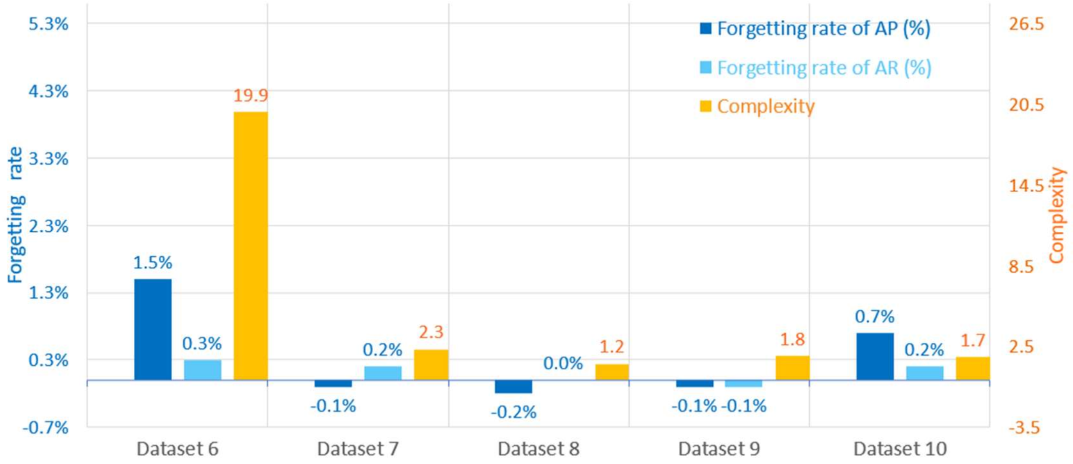

Supplementary Figure 2. **Relation between complexity of new dataset and forgetting rate on old datasets.** The complexity (yellow) and the forgetting rate of AP (blue) and AR (light blue) for sequentially added new dataset from dataset 6 to dataset 10 were shown. Dataset 6 (Beta-galactosidase) has the highest complexity, which does have very different features from dataset 1~5 (see the second row in **Table 1**). While the high complexity of dataset 6 causes a forgetting rate up to 1.5%, the forgetting rate is still minor compared with the absolute AP value of ~90%. The low complexities of dataset 7~10 cause subtle forgetting, and even improve the performance of picking the old datasets (Dataset 7~9, indicated by the negative forgetting rates).

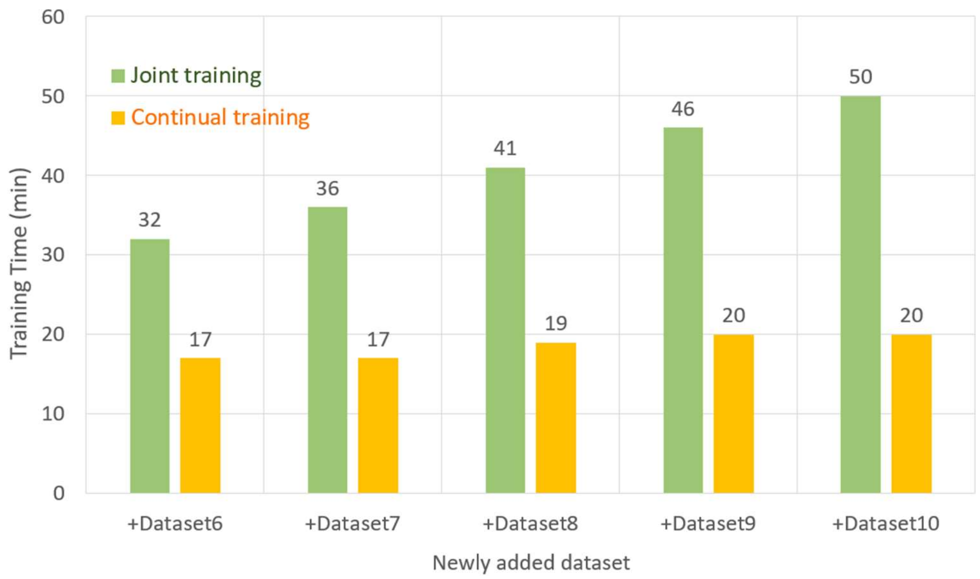

Supplementary Figure 3. **Comparison of training time between continual learning and joint training.** An initial model was trained on micrographs from five EMPIAR datasets: 1–5. Then, EMPIAR dataset 6 to dataset 10 were added one by one. The training time for joint training increases linearly with the addition of new datasets. However, the training time for continual learning changes slightly with the increase of samples in the exemplar dataset.

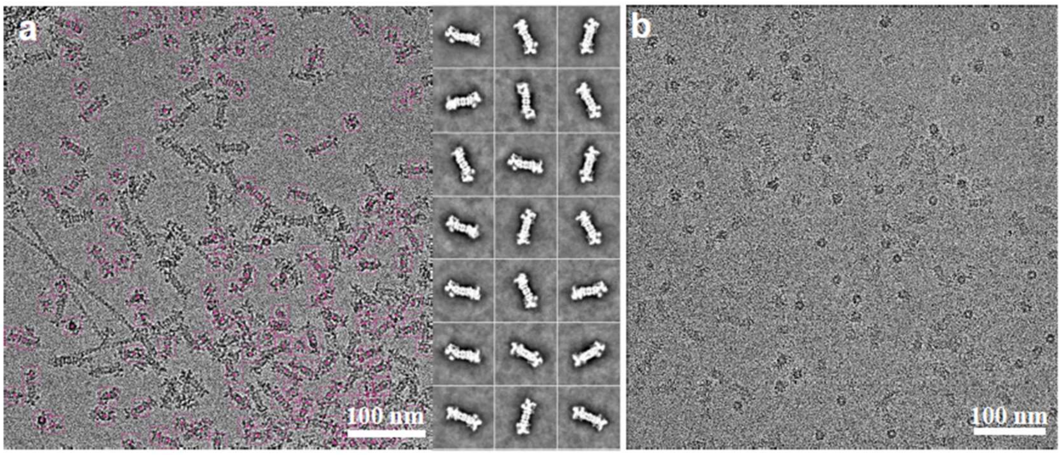

Supplementary Figure 4. Typical micrographs of 26S datasets. a) A typical micrograph in the EMPIAR-10090 dataset and typical 2D class averages of the side-view CP2RP particles used for training. In total, 12366 particles were picked from 114 randomly selected micrographs using EPicker with the general model trained on 46 datasets without 26S and 20S proteasomes. The picked particles are labeled with boxes. Finally, 9651 CP2RP particles were selected by 2D class averages. **b**) A typical micrograph in EMPIAR-10401. The two datasets were acquired under 200 and 300 kV voltage, respectively, and have different contrast and purity.

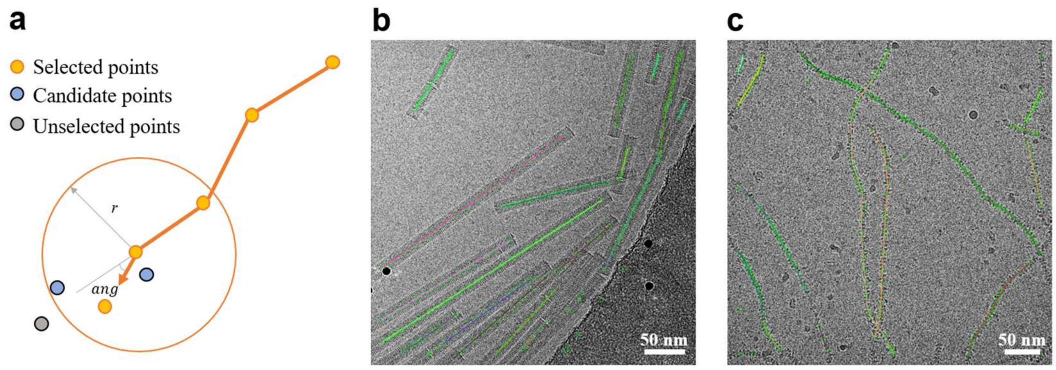

Supplementary Figure 5. Fiber tracing algorithm. a) Illustration of the line tracing algorithm (LTA). The LTA selects candidate points according to radius r and angle ang. **b**) A typical micrograph in EMPIAR-10020. **c**) A typical micrograph from EMPIAR-10211, corresponding to **Fig. 5a**. The initial picking of fiber segments are shown as green boxed, and the tracing results are shown as lines with different colors.

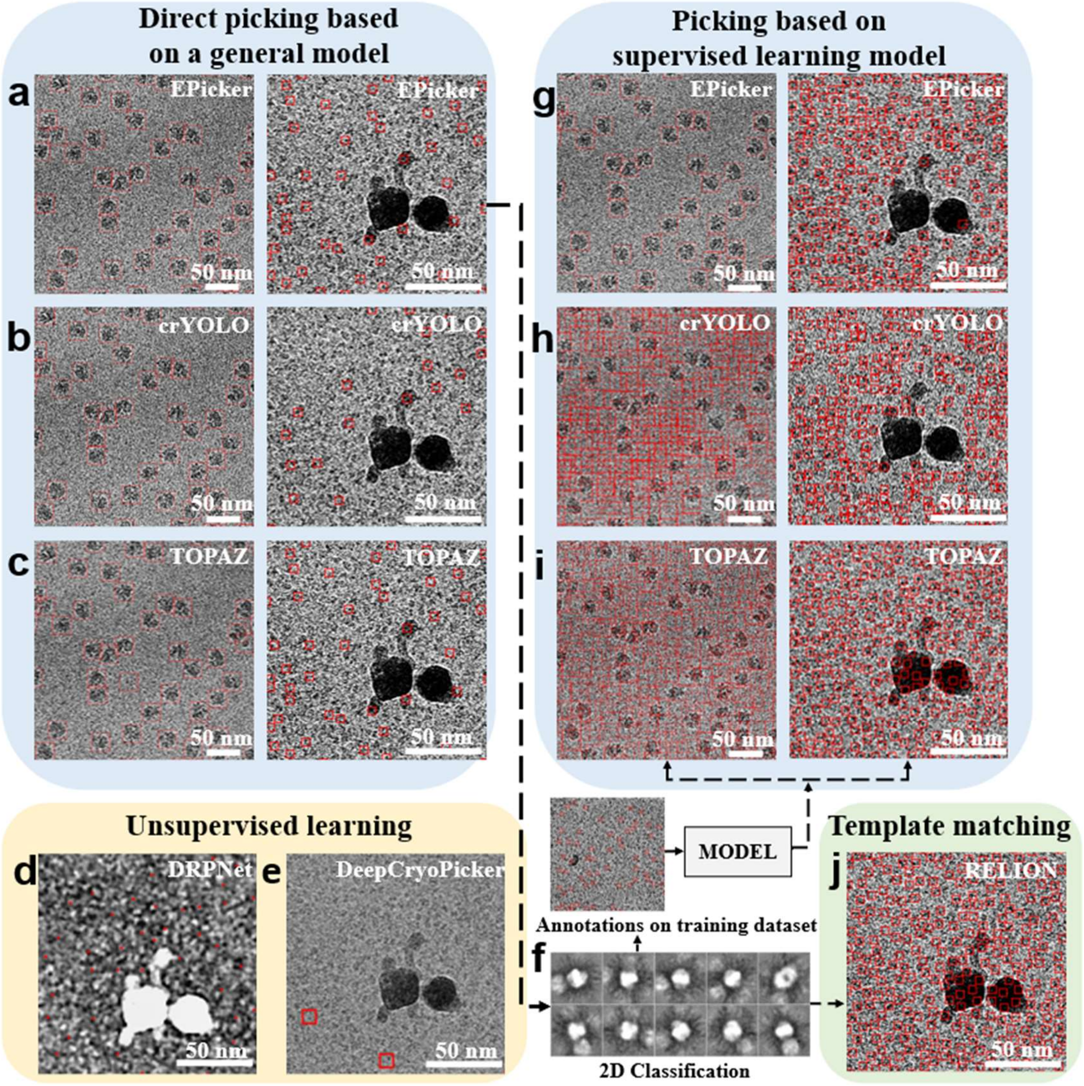

Direct picking based on a general model
Picking based on supervised learning model
a
EPicker
b
crYOLO
c
TOPAZ
g
EPicker
h
crYOLO
i
TOPAZ
50 nm
Unsupervised learning
d
DRPNet
e
DeepCryoPicker
MODEL
Annotations on training dataset
f
2D Classification
Template matching
j
RELION

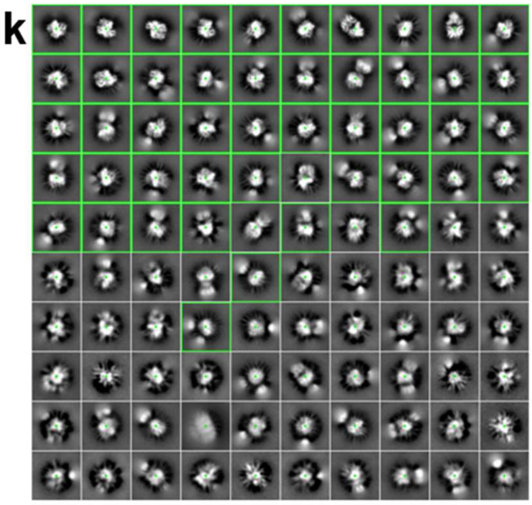

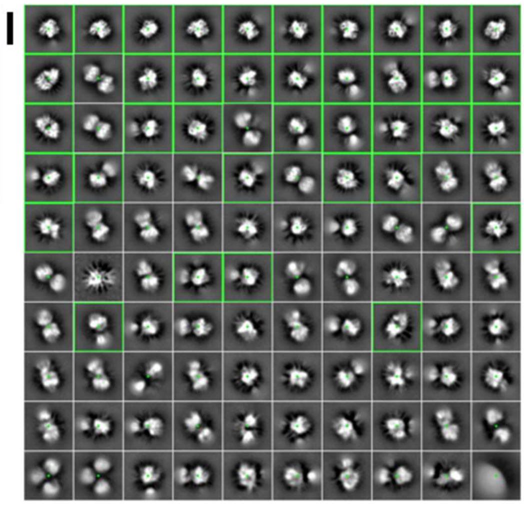

EPicker
(68.2%, 397191 of 582326 particles in 47 classes)

crYOLO
(52.6%, 303837 of 578114 particles in 38 classes)

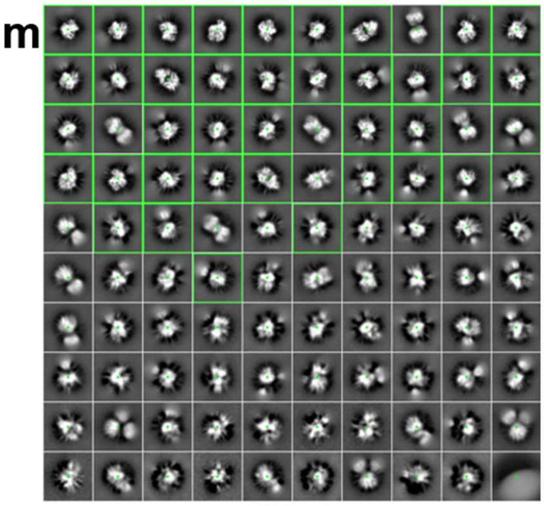

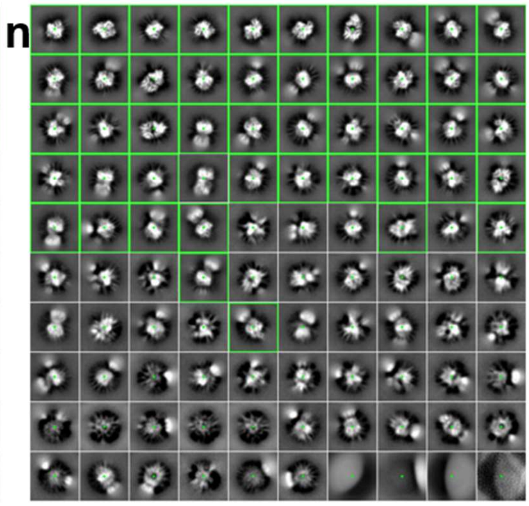

TOPAZ
(57.1%, 337530 of 590731 particles in 38 classes)

RELION
(67.9%, 387072 of 569993 particles in 47 classes)

Supplementary Figure 6. Comparison of different software. A Fab dataset with 179 micrographs were used to evaluate different software. A ribosome micrograph was also used. The Fab rarely appeared in public databases, and hence is a good "unknown" sample for most published software. In contrast, the ribosome dataset is contained in most of public databases, and thus is a "known" sample for the published software. **a–c)** Typical picking results on the ribosome and Fab micrographs of EPicker, crYOLO[6], and TOPAZ[5], respectively. The general model of EPicker was trained on 50 datasets excluding Fab (**Supplementary Table 9**). The general model of crYOLO was trained on a combination of 53 datasets. And the number of datasets used to train the general model of TOPAZ is unknown. All three software show ideal performance on the ribosome, but not ideal on the Fab. **d-e)** Picking results of DRPNet[7] and DeepCryoPicker[8] on Fab. Both software employed unsupervised-learning algorithms and failed to pick Fabs. No further tests can be performed since a training procedure is not available. **f)** Ten selected 2D classes and corresponding particle annotations of Fab. To generate a training dataset, the initial picking results of EPicker (shown in **a**) on 8 micrographs in the Fab dataset were filtered by THUNDER[9] 2D classification. In the

ten classes, 6562 particles showing obvious features of the Fab were selected as training annotations and then were used to build the training dataset for EPicker, crYOLO and TOPAZ. The ten class averages were used as the templates for RELION[10]. **g–i)** Typical picking results on the ribosome and Fab micrographs of EPicker, crYOLO, and TOPAZ, respectively, after loading the corresponding general models and training on the newly built Fab dataset. The continual learning was used for EPicker and the fine-tuning was used for crYOLO and TOPAZ. All these three software performed as expected on Fab. For crYOLO and TOPAZ, the performance on the ribosome dataset was not maintained, indicated by lots of missed picking on the ribosome. While the picking results of EPicker were similar to those before the training on Fab. **j)** A typical picking result of RELION based on template matching using the ten class averages. RELION cannot avoid picking ice particles and hence did not perform as well as the other three deep-learning-based software. **k–n**) The results of 2D classification on the picking results of EPicker, crYOLO, TOPAZ, and RELION, corresponding to the processing in **g**–**j**. All software picked ~600,000 particles (details shown on the bottom of each figure). After empirically selecting classes well centered and with secondary-structure details, EPicker and RELION output more good particles (~390,000) than the other two software.

Supplementary References:

1. Aljundi, R., Babiloni, F., Elhoseiny, M., Rohrbach, M. & Tuytelaars, T. Memory Aware Synapses: Learning what (not) to forget. in *Proceedings of the* European *conference on computer vision* 139-154 (2017).
2. Peng, C., Zhao, K. & Lovell, B.C. Faster ILOD: Incremental learning for object detectors based on faster RCNN. *Pattern Recognition Letters* **140**, 109-115 (2020).
3. K., H., X., Z., S., R. & J., S. Deep residual learning for image recognition in *IEEE Conference on* Computer Vision *and* Pattern Recognition *(CVPR)* 770-778 (2016).
4. Yu, F., Wang, D., Shelhamer, E. & Darrell, T. Deep layer aggregation. in *NIPS Workshop*(2017).
5. Bepler, T. et al Positive-unlabeled convolutional neural networks for particle picking in cryo-electron micrographs. *Nature Methods* **16**, 1153-1160 (2019).
6. Wagner, T. et al SPHIRE-crYOLO is a fast and accurate fully automated particle picker for cryo-EM. *Communications Biology* **2**, 218-218 (2019).
7. Nguyen, N.P., Ersoy, I., Gotberg, J. et al. DRPnet: automated particle picking in cryo-electron micrographs using deep regression. *BMC Bioinformatics* **22**, 55 (2021).
8. Al-Azzawi, A., Ouadou, A., Max, H. et al. DeepCryoPicker: fully automated deep neural network for single protein particle picking in cryo-EM. *BMC Bioinformatics* **21**, 509 (2020).
9. Hu, M. et al A particle-filter framework for robust cryo-EM 3D reconstruction. *Nature Methods* **15**, 1083-1089 (2018).
10. Scheres, S.H.W. RELION: Implementation of a Bayesian approach to cryo-EM structure determination. *Journal of Structural Biology* **180**, 519-530 (2012).
